# Diagnosis, treatment, and prognosis of primary intraocular lymphoma: Single‐center real‐world clinical experience

**DOI:** 10.1002/cam4.5567

**Published:** 2023-01-31

**Authors:** Gi‐June Min, Tong Yoon Kim, Young‐Woo Jeon, Joo Hyun O, Byung‐Ock Choi, Gyeongsin Park, Young‐Hoon Park, Seok‐Goo Cho

**Affiliations:** ^1^ Department of Hematology, Catholic University Lymphoma Group Seoul St. Mary's Hospital, College of Medicine, The Catholic University of Korea Seoul Republic of Korea; ^2^ Department of Hematology, Catholic University Lymphoma Group Yeouido St. Mary's Hospital, College of Medicine, The Catholic University of Korea Seoul Republic of Korea; ^3^ Department of Nuclear Medicine, Catholic University Lymphoma Group Seoul St. Mary's Hospital, College of Medicine, The Catholic University of Korea Seoul Republic of Korea; ^4^ Department of Radiation Oncology, Catholic University Lymphoma Group Seoul St. Mary's Hospital, College of Medicine, The Catholic University of Korea Seoul Republic of Korea; ^5^ Department of Hospital Pathology, Catholic University Lymphoma Group Seoul St. Mary's Hospital, College of Medicine, The Catholic University of Korea Seoul Republic of Korea; ^6^ Department of Ophthalmology and Visual Science, Catholic University Lymphoma Group Seoul St. Mary's Hospital, College of Medicine, The Catholic University of Korea Seoul Republic of Korea

**Keywords:** CNS relapse, local intravitreal methotrexate, primary intraocular lymphoma, systemic high‐dose methotrexate

## Abstract

**Background:**

The diagnosis and management of primary intraocular lymphoma (PIOL) remain challenging. This study identified factors indicative of PIOL, described treatment outcomes, and determined modalities to prevent relapse.

**Methods:**

We included 21 PIOL‐diagnosed patients, seven via cytology, 12 via genetic evaluation, and two via interleukin (IL) level measurements, who underwent vitrectomy and received local intravitreal methotrexate (IV‐MTX) injection. Clinical outcomes, including treatment response and relapse, were compared between patients receiving IV‐MTX alone (n = 13) or IV‐MTX with systemic high‐dose methotrexate (HD‐MTX) as prophylaxis (n = 8).

**Results:**

Twelve ophthalmologic and eight central nervous system (CNS) relapse cases within a median of 20.3 and 11.6 months were shown, regardless of the treatment modalities, with a median progression‐free survival of 21.3 (95% confidence interval, 9.5–36.7) months. There was no difference in demographic characteristics between the two groups, except with the poorer performance status in patients in the HD‐MTX prophylaxis group. Furthermore, patients demonstrated rapid elevations in the vitreous fluid IL‐10/IL‐6 cytokine ratio before ophthalmologic and CNS relapse. Therefore, diagnosis should be based on clinical signs and assisted by vitrectomy, cytologic, molecular, and cytokine studies.

**Conclusion:**

For PIOL, aggressive systemic treatment equivalent to that of primary CNS lymphoma (PCNSL) is recommended because solely HD‐MTX did not prevent or delay CNS relapse. To prevent PIOL relapse in the CNS efficiently, prospective trials with large numbers of patients and advanced therapeutic regimens are necessary. Furthermore, regular clinical follow‐up is crucial, and the IL‐10/IL‐6 ratio can help evaluate relapse promptly.

## Graphical abstract

For primary intraocular lymphoma patients, IL‐10/IL‐6 cytokine evaluation for diagnosis and relapse monitoring should be routine practice, and clinicians may consider more intensive or novel therapeutic regimens for CNS prophylaxis.

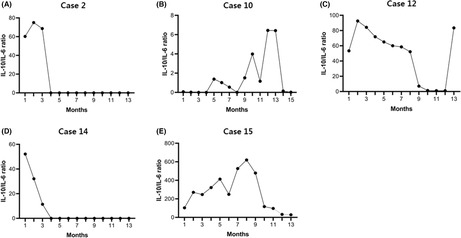

## 1 INTRODUCTION

Primary intraocular lymphoma (PIOL) is a subset of primary central nervous system lymphoma (PCNSL) characterized by intraocular involvement without evidence of another disease in the brain or cerebrospinal fluid.^1^, ^2^, ^3^ PIOL only accounts for approximately 5% of primary brain tumors and 1% of extranodal lymphomas; thus, its exact epidemiology is unknown.^4^, ^5^, ^6^ Approximately, one‐third of PIOL patients will have concurrent PCNSL at presentation, and a further 42%–92% will eventually develop PCNSL.^1^, ^7^, ^8^, ^9^, ^10^, ^11^ Furthermore, CNS progression could develop within months after the onset of visual symptoms from PIOL.^4^, ^11^, ^12^, ^13^ Therefore, once PIOL is diagnosed, neurologic staging according to the AJCC/UICC TNM Classification via magnetic resonance imaging (MRI) and 18‐fluorodeoxyglucose positron emission tomography (FDG‐PET) imaging and spinal tapping should be performed to exclude other CNS lesions. Secondary intraocular lymphoma is the infiltration of lymphoma developed from outside of the CNS and must be differentiated from PIOL. PIOL can spread into the vitreous, retina, retinal pigment epithelium, Bruch's membrane, or optic nerve and accounts for up to 95% of diffuse large B‐cell lymphoma (DLBCL).^1^, ^2^, ^3^ Any disease‐causing choroidal, retinal, subretinal, or vitreous chamber infiltration could mimic or be mimicked by PIOL. Because PIOL can “masquerade” as uveitis of various causes, the diagnosis of PIOL remains challenging, and differential diagnosis should cover all types of chronic posterior uveitis.^8^, ^14^ The hallmark of PIOL is the presence of vitreal cells, mainly in clumps. Therefore, diagnostic vitrectomy should involve cytologic analysis of vitreal samples for the existence of malignant cells. The additional use of immunocytochemistry, flow cytometry, and cytokine profile evidence (interleukin [IL]‐10 to IL‐6 ratio >1.0) from vitreous fluid markedly helps laboratory diagnosis of PIOL via cytology.^1^, ^15^ This study aimed to identify factors indicative of PIOL to improve diagnosis accuracy, describe treatment outcomes, and determine effective modalities for preventing relapse.

## 2 METHODS

### 2.1 Study design and patients

The subjects were 24 consecutive patients diagnosed with PIOL and followed up between October 2013 and March 2020. All 24 patients underwent vitrectomy and local intravitreal methotrexate (IV‐MTX) injection for vitreous opacity at Seoul St. Mary's Hospital. The procedure was performed by a skilled ophthalmologist with more than 10 years of experience in vitreous surgery. This retrospective study was approved by the Institutional Review Board and Ethics Committee of the Catholic Medical Center, Republic of Korea (KC21RASI0761) and conducted according to the tenets of the Helsinki Declaration. The need for informed consent was waived owing to the retrospective nature of the study.

### 2.2 Diagnostic protocol

PIOL was diagnosed based on the combined results of several tests. Cytologic examinations for initial pathologic diagnosis were conducted using vitreous samples obtained via pars plana vitrectomy or vitreal fine‐needle aspiration.^4^, ^16^ Assessing the presence of malignant PIOL cells via cytopathology with immunohistochemical staining is currently the most reliable diagnostic indicator [Diagnosis level I]. However, when the concentration of neoplastic cells was inadequate, flow cytometry and molecular studies such as polymerase chain reaction (PCR) to assess immunoglobulin heavy chain rearrangement and monoclonality were performed to identify lymphoma cells. Both flow cytometry and PCR can locate lymphoma cells and present monoclonality with mature B‐cell markers and/or light chain restriction [Diagnosis level II].^1^, ^4^, ^8^ However, we aided the diagnosis of PIOL using IL‐10/IL‐6 level analysis via enzyme‐linked immunosorbent assay (ELISA) when the cytologic or molecular evaluation still showed ambiguous results. Cytokines IL‐10 and IL‐6 were utilized to discriminate PIOL and ocular inflammatory processes.^1^, ^14^ An IL‐10 to IL‐6 ratio of >1 indicated a potential diagnostic biomarker for PIOL [Diagnosis level III].^1^, ^15^, ^17^

PIOL patients were further evaluated for concurrent CNS lesions or systemic involvement. At the initial diagnosis, we performed neck, chest, and abdomen/pelvic computed tomography (CT) and brain and torso FDG‐PET/CT for staging workup. Gadolinium‐enhanced MRI was also performed to evaluate concurrent CNS lesions. All patients also underwent laboratory, comprehensive physical and neurologic examinations. We excluded HIV‐positive patients in this study. An ophthalmologic evaluation was performed at diagnosis, response workup, and follow‐up to visualize ocular involvement. Ophthalmologic follow‐up evaluations were performed every 3–6 months according to the clinician's discretion.

### 2.3 Treatment strategies

All patients received local IV‐MTX injections at regular intervals after PIOL was diagnosed, although the vitreous IL‐10/IL‐6 cytokine ratio workup and IV‐MTX injection as a routine procedure became established after 2018. Local IV‐MTX treatment included an intravitreal injection of methotrexate (400 μg/0.1 ml) twice weekly for 8 weeks for the induction phase, once weekly for the subsequent 8 weeks for the consolidation phase, and once monthly for 9 months for the maintenance phase. In total, 25 injections were given in 1 year.^18^ IV‐MTX was injected at the pars plana level using a 30‐gauge needle under topical anesthesia with tetracaine HCl 0.5% drops. The patient's visual acuity and intraocular pressure (IOP) were evaluated using dilated fundoscopy to assess malignant cellular infiltrates in the retina and/or optic nerve head. In addition, slit‐lamp biomicroscopy was conducted to view cells within the vitreous cavity in each visit. All patients diagnosed with PIOL without concurrent CNS lesions planned to receive both local therapeutic IV‐MTX and prophylactic systemic high‐dose methotrexate (HD‐MTX) systemic therapy. However, 13 patients (54.2%) did not receive HD‐MTX because either the patient refused treatment or the clinician did consider the patient's poor performance status or renal dysfunctions.

Prophylactic systemic HD‐MTX treatment was initiated when local IV‐MTX injection was administered routinely every month. A total of 12 cycles of 1‐day HD‐MTX (3.5 g/m^2^) intravenous infusion with 2‐week intervals was administered on an inpatient basis for 6 months. Because methotrexate is primarily excreted via the kidneys, drug elimination becomes problematic once nephrotoxicity occurs. HD‐MTX could cause lethal adverse effects, and thus, the blood methotrexate level was closely monitored. Leucovorin rescue was promptly provided as needed. Along with the dose‐adjusted leucovorin rescue protocol, prophylactic strategies included routine monitoring of the blood concentration of MTX and creatinine level and aggressive hydration. Leucovorin (50 mg/m^2^) was administered intravenously every 6 h. The blood methotrexate concentration was monitored 24 h after HD‐MTX infusion was initiated. Serial 24‐, 48‐, and 72‐h blood concentrations of methotrexate should be below 5.0 μM/L, 0.5 μM/L, and 0.05 μM/L, respectively. If the serum creatinine increased above 50% from baseline or the blood methotrexate concentration did not decrease below the established level in time, the fluid infusion was increased to 200 ml/h; leucovorin (100 mg/m^2^) was adjusted and administered every 3 h, and the blood methotrexate concentration was closely monitored.

Patients with PIOL with concurrent CNS lesions at diagnosis or CNS relapse received the standard PCNSL treatment protocol of our institute, which included six cycles of single HD‐MTX (3.5 g/m^2^) with 2‐week intervals and three cycles of a combination regimen of HD‐MTX (3.5 g/m^2^) for 1 day and subsequent cytosine arabinoside (ARA‐C/cytarabine; 3.0 g/m^2^) intravenous infusion for 2 days with 4‐week intervals with or without whole‐brain radiotherapy (WBRT). However, considering the complications of cytopenias, we reduced the dose of cytarabine in elderly patients older than 60 years. Furthermore, we decided not to use cytarabine in patients either with age > 65 years with poor performance (Eastern Cooperative Oncology Group performance status [ECOG PS] ≥ 2) or with underlying chronic renal failure. Patients with CNS relapse who achieved complete remission (CR), unconfirmed CR (uCR), or partial remission (PR) after standard PCNSL treatment, whose age was ≤65 years, and whose ECOG PS was 0–2 were selected for autologous hematopoietic stem cell transplantation (auto‐HSCT). We used a TBC regimen (thiotepa, busulfan, and cyclophosphamide) with a 20% reduction in both thiotepa and cyclophosphamide of the previous dose for auto‐HSCT.^19^

### 2.4 Definitions for response evaluation

Treatment response was evaluated via ocular examination according to the response evaluation criteria for PCNSL. CR was defined as normal in the ocular examination; uCR as minor abnormality in the retinal pigment epithelium; PR as a decrease in retinal infiltrate or vitreous cells; and progressive disease as recurrent or new ocular disease.^20^ The clinical response to IV‐MTX, including indicators of CR (e.g., the absence of cells from the vitreous and absence/minimal of retinal and optic nerve head infiltrates), was routinely evaluated by ophthalmologists. We defined relapse as ophthalmologic and CNS relapse. Ophthalmologic relapse was diagnosed when patients met more than two of the three criteria: (a) visual symptoms, (b) vitreous cell clusters on ophthalmologic routine follow‐up examination to restart IV‐MTX injection, or (c) a rapidly elevated IL‐10/IL‐6 ratio >1 in the vitreous fluid. CNS relapse was diagnosed when CNS lymphoma involvement was confirmed via CT, FDG‐PET/CT, or MRI with pathologic evaluation.

### 2.5 Statistical analysis

All categorical variables were compared using the Chi‐square or Fisher's exact test and continuous variables, the Student's *t*‐test or the Mann–Whitney *U* test for comparisons between two groups. Overall survival (OS) and progression‐free survival (PFS) were estimated using the Kaplan–Meier method. OS was defined as the time from diagnosis of PIOL to death or last follow‐up, and PFS was calculated from diagnosis to disease progression, relapse after CR, or death. The cumulative incidence of relapse (CIR) was calculated by cumulative incidence estimation, for which non‐relapsed mortality was considered a competing risk, and all results were compared using the Gray test. Multivariate analysis was performed using stepwise selection among candidate variables chosen from univariate analysis and excluding highly correlated variables. Fine‐Gray proportional hazard regression model was used to calculate the hazard ratios for cumulative incidences of CNS relapse. The censor date for our data was either until the last follow‐up day of the patient or the patient's day of death. Statistical significance was set at a *p*‐value of <0.05 (2‐sided), and the statistical analyses were performed using the “R” software version 3.4.1 (R Foundation for Statistical Computing, 2017).

## 3 RESULTS

### 3.1 Baseline patient characteristics

**FIGURE 1:**
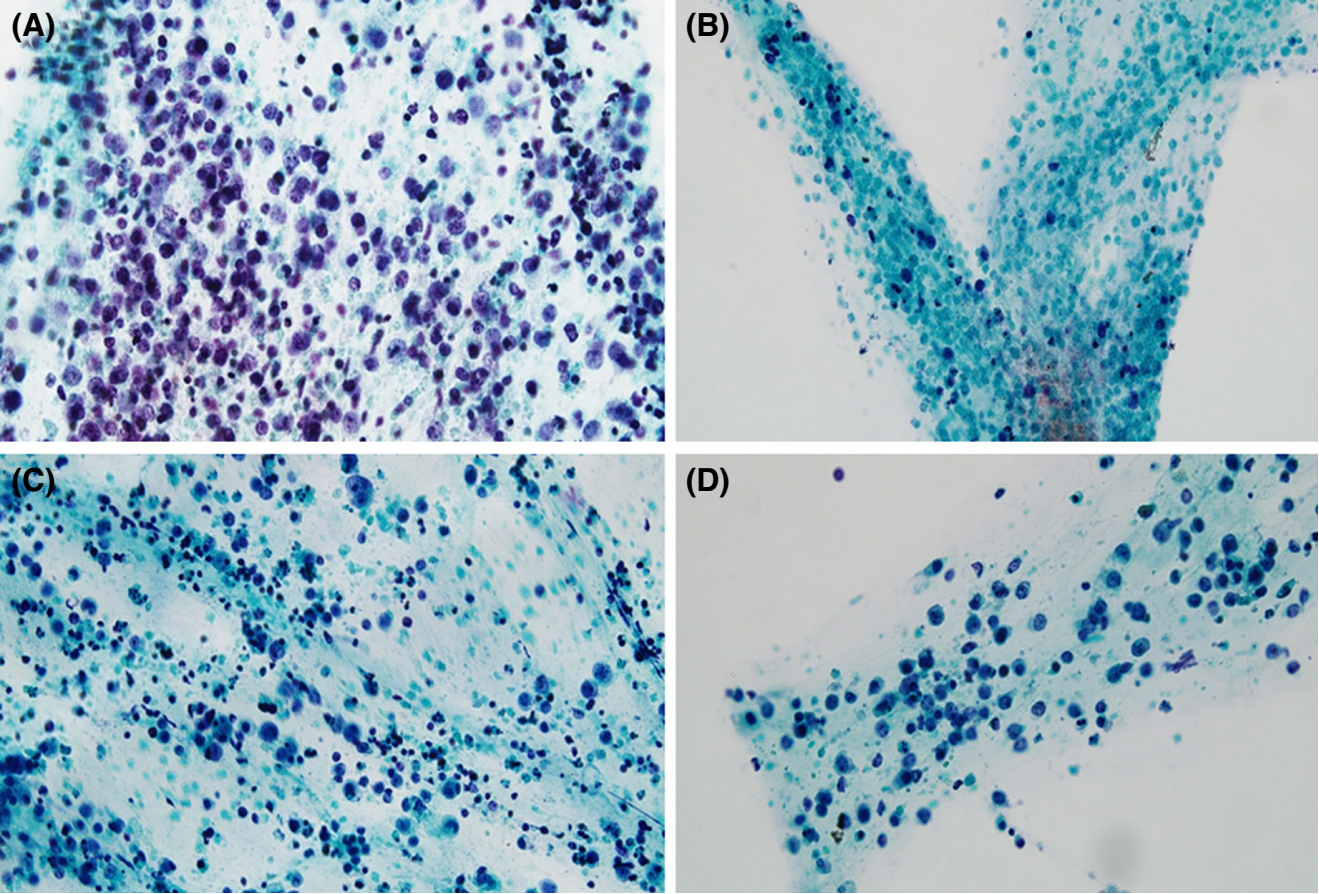
Cytology of a vitreous specimen from patients with PIOL for Cases 9 (A), 10 (B), 15 (C), and 16 (D) showing large atypical lymphoid cells with scanty basophilic cytoplasm and large irregular nuclei. (Papanicolau stain, ×400). PIOL, primary intraocular lymphoma.

Of the 21 patients with PIOL, 11 were female and 10 male. The median age was 65.0 (range: 32.0–81.0) years. The median interval between the onset of ocular symptoms and the diagnosis of PIOL was 10.8 (range: 1.7–30.6) months. PIOL was diagnosed via confirmative cytology assisted with immunohistochemical staining in seven patients (33.3%) (Diagnosis level I); via additional flow cytometry and molecular studies in 12 (57.2%) (Diagnosis level II); and via IL‐10/IL‐6 cytokine level measured using ELISA with clinical evidence in 2 (9.5%) (Diagnosis level III). Cytologic diagnosis of PIOL is based on a combination of morphology and immunohistochemistry and requires an appropriate quantity of the sample. These seven patients, with diagnosis level I, presented with atypical lymphocyte with large sizes (more than 2 times), and scanty basophilic cytoplasm, large irregular nuclei, and prominent nucleoli (Figure 1). When it was inappropriate to use a vitreous sample for cytologic diagnosis, flow cytometry and molecular studies were performed to detect B‐cell lymphoma populations and/or B‐lymphocyte monoclonality. Eight patients with analyzable lymphoma cell populations showed predominant CD19‐positive (> 90%) B cell with kappa clone during flow cytometry. However, clonal IgH and Ig kappa rearrangement analysis were positive in all 12 patients at diagnosis level II. Although IL‐10/IL‐6 cytokine ELISA was routinely performed during follow‐up, during diagnosis, it was only performed in two cases. The diagnosis in these two cases depended on cytokine level due to an inadequate amount of vitreous sample to perform cytologic or molecular diagnoses.

Twelve patients (57.1%) were previously treated for anterior, intermediate, or posterior uveitis, pars planitis, macular degeneration, or postoperative complications of cataract surgery before PIOL was diagnosed. Among them, nine patients were treated with intravenous steroid injection and oral steroid maintenance under the impression of anterior, intermediate, posterior, or panuveitis. Two of them used adalimumab as a second‐line agent to treat uveitis. Oral corticosteroid maintenance showed only temporal improvement of floaters or other visual disturbances. All enrolled patients showed negative CSF study at the time of PIOL diagnosis. Table 1 summarizes the baseline patient characteristics and treatment courses.

**TABLE 1:** Characteristics and clinical outcomes of primary intraocular lymphoma (*N* = 21)

Characteristics	Values
Age, median (range)	65.0 (32.0–81.0) years
Sex, female and male	11 (52.4%) and 10 (47.6%))
Pretreated patients^a^	12 (57.1%)
From symptom onset to diagnosis, median (range)	10.8 (1.7–30.6) months
Diagnosis level	
Cytologic diagnosis [level I]	7 (33.3%)
Flow cytometry and immunohistochemistry [level II]	12 (57.2%)
Cytokine (IL‐10/IL‐6 ratio) + clinical evidence [level III]	2 (9.5%)
Diagnosis lesions	
Left vitrectomy	9 (42.9%)
Right vitrectomy	10 (47.6%)
Both vitrectomy	2 (9.5%)
Initial treatment	
IV‐MTX only	13 (61.9%)
IV‐MTX and HD‐MTX	8 (38.1%)
Ocular complication due to IV‐MTX	
Corneal epitheliopathy	7 (33.3%)
Glaucoma, uncontrolled	3 (14.3%)
Cataract, required surgery	3 (14.3%)
Macular edema and vitreous hemorrhage	1 (4.8%)
Blepharitis	1 (4.8%)
Limbal ischemia	1 (4.8%)
Ocular response	
Complete remission	15 (71.4%)
Unconfirmed complete remission	3 (14.3%)
Partial remission	3 (14.3%)
Follow‐up period, median (range)	27.9 (6.8–68.6) months
Relapse	
Ophthalmologic relapse, median (range)	*N* = 12, 20.3 (4.7–53.4) months
Ipsilateral relapse	6 (50.0%)
Contralateral relapse	1 (8.3%)
Both side relapse, oculi uterque	5 (41.7%)
CNS relapse, median (range)	*N* = 8, 11.6 (8.8–26.7) months
Salvage systemic therapy (*n* = 8)	
PCNSL protocol	5 (62.5%)
Subsequent autologous HSCT	1 (20.0%)
HD‐MTX	1 (12.5%)
WBRT	1 (12.5%)
R‐CHOP 100%^b^	1 (12.5%)

Abbreviations: CNS, central nervous system; HD‐MTX, high‐dose intravenous methotrexate; HSCT, hematopoietic stem cell transplantation; IL, interleukin; IV‐MTX, intravitreal methotrexate; R‐CHOP, consists of rituximab; cyclophosphamide; doxorubicin, vincristine; and prednisone; WBRT, whole‐brain radiotherapy.

^a^ Twelve patients (57.1%) treated as anterior/intermediate/posterior uveitis, pars planitis, macular degeneration, or postoperative complication of cataract surgery before PIOL was diagnosed.

^b^ One patient relapsed as systemic DLBCL involvement (Ann‐Arbor stage IV), including CNS, required R‐CHOP chemotherapy.

### 3.2 Clinical responses and treatment outcomes

The median follow‐up period was 27.1 (range: 6.8–68.6) months. The 5‐year estimated OS was 92.9% (95% confidence interval [CI], 59.1–99.0). However, the 5‐year estimated PFS was 0% due to delayed ocular recurrence events at 53.3 months after treatment, and the median PFS was 21.3 months (95% CI, 9.5–36.7). The CIR of the ophthalmologic (*n* = 12) and CNS lesion (*n* = 8) was 89.5% (95% CI, 62.3–97.4) and 42.0% (95% CI, 19.4–63.1), respectively (Figure S1).

**FIGURE 2:**
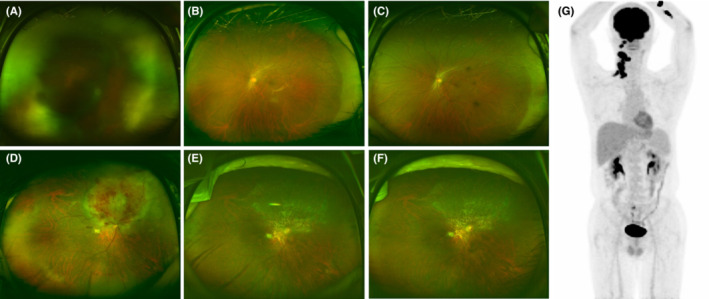
Clinical cases of PIOL diagnosis and treatment. (A and B) Fundus photographs of Case 3 at baseline and 3 months after IV‐MTX treatment. (C) Case 3 patient maintained complete remission by fundus photographs and IL‐10/IL‐6 ratio <1 by cytokine panel. (D and E) Fundus photographs of Case 11 at baseline and 6 months after IV‐MTX and HD‐MTX combined treatment. (F) Despite improvement in fundus photographs, IL‐10/IL‐6 ratio rose (>1) at 5.6 months, and systemic relapse was diagnosed at 11.8 months after achieving the first PR. (G) Case 11 relapsed as systemic diffuse large B cell lymphoma active B‐cell type, confirmed by pathologic specimen of the nasopharyngeal mass as Ann‐Arbor stage IV, based on FDG‐PET CT, and received R‐CHOP chemotherapy. FDG‐PET–CT, fluorodeoxyglucose (FDG)‐positron emission tomography (PET)–computed tomography (CT); HD‐MTX, high‐dose methotrexate; IL, interleukin; IV, intravenous; PIOL, primary intraocular lymphoma; R‐CHOP, rituximab (R) cyclophosphamide (C) doxorubicin (H) vincristine (O) prednisolone (P).

Table 2 shows the detailed clinical information of all patients. Six patients (Cases 1–6) did not experience relapse after initial treatment and have since maintained CR. Eight patients (Cases 7–14) developed a CNS lesion within a median of 11.6 (range, 8.8–26.7) months after achieving CR (*n* = 3), uCR (*n* = 3), or PR (*n* = 2) of PIOL to local IV‐MTX injection and/or systemic HD‐MTX infusion. Most patients with CNS relapse were treated based on the PCNSL treatment protocol. One patient (Case 12) underwent auto‐HSCT soon after achieving CR, using the PCNSL treatment protocol, and is still alive. However, two elderly patients, one with renal impairment (Case 7) and one with poor performance status (Case 13) after relapse, were unable to use cytarabine; thus, they mainly received salvage WBRT or HD‐MTX only. Case 13 died during salvage HD‐MTX because of septic shock. The remaining cases (Cases 7, 8, 9, 10, 11, 12, and 14) achieved a second CR (*n* = 5) or PR (*n* = 2) and are still alive. One patient (Case 11) developed systemic relapse as DLBCL, including a CNS lesion, 11.8 months after PIOL was diagnosed. This patient further underwent R‐CHOP chemotherapy consisting of cyclophosphamide (750 mg/m^2^), doxorubicin (50 mg/m^2^), vincristine (1.4 mg/m^2^), and rituximab (375 mg/m^2^) on day 1, and oral prednisone (60 mg/m^2^) on days 1 to 5 every 21 days. We presented fundus photographs findings before and after treatment of Case 3 and Case 11 and FDG‐PET CT findings after the relapse of Case 11 in Figure 2. Among the 12 patients (Cases 10–21) with ophthalmologic relapse, the tumor developed on the same (*n* = 6) or opposite (*n* = 1) side of the initial ocular lesion or both (*n* = 5) within a median of 20.3 (range: 4.7–53.4) months, which indicated distant rather than systemic relapse of median 11.6 (range: 8.8 to 26.7) months. Seven patients (Cases 15–21) presented with isolated ophthalmologic relapse and achieved CR (*n* = 6) or PR (*n* = 1) after salvage IV‐MTX only. Five patients (Cases 10–14) experienced both ophthalmologic and CNS relapses consecutively; these patients received salvage therapy, three of whom achieved CR.

**TABLE 2:** Summary of the clinical course of the primary intraocular lymphoma patients (*N* = 24)

Case	Sex/age (years)	Symptom to diagnosis (mo)	Vitrectomy	Diagnosis evidence	Concurrent CNS lesion	Initial treatment	Complications	Response	Ophthalmologic Relapse (from response)	CNS relapse (from response)	Clinical outcomes (follow‐up period)
1	F/60	5.0	Right	II	No	IV‐MTX		CR	**No**	**No**	Alive (10.8 mo)
2	M/52	16.7	Right	III	No	IV‐MTX + HD‐MTX	Glaucoma	CR	**No**	**No**	Alive (7.8 mo)
3	M/32	10.8	Left	II	No	IV‐MTX	Cataract	CR	**No**	**No**	Alive (30.2 mo)
4	M/56	14.6	Left	II	No	IV‐MTX + HD‐MTX	Macular edema and Vitreous hemorrhage	CR	**No**	**No**	Alive (37.5 mo)
5	F/75	24.0	Right	I	No	IV‐MTX		CR	**No**	**No**	Alive (6.8 mo)
6	M/81	2.7	Right	II	No	IV‐MTX	Corneal epitheliopathy	CR	**No**	**No**	Alive (15.6 mo)
7	F/67	4.1	Right	II	No	IV‐MTX	Glaucoma	uCR	No	Yes (8.8 mo)	Salvage WBRT, PR, Alive (12.4 mo)
8	M/70	13.0	Left	II	No	IV‐MTX		CR	No	Yes (9.5 mo)	PCNSL protocol, CR Alive (35.5 mo)
9	M/55	2.0	Both	I	No	IV‐MTX + HD‐MTX	Limbic ischemia	CR	No	Yes (18.6 mo)	PCNSL protocol with auto‐HSCT, CR, Alive (27.9 mo)
10	M/72	1.7	Right	I	No	IV‐MTX + HD‐MTX	Corneal epitheliopathy	PR	Yes (4.7 mo) Both	Yes (11.3 mo)	PCNSL protocol, PR Alive (18.0 mo)
11	M/54	12.8	Right	III	No	IV‐MTX + HD‐MTX	Corneal epitheliopathy	PR	Yes (5.4 mo) Both	Yes (11.8 mo)	Salvage R‐CHOP^a^, CR Alive (17.1 mo)
12	F/65	4.4	Left	II	No	IV‐MTX + HD‐MTX	Corneal epitheliopathy	uCR	Yes (15.7 mo) Left	Yes (26.7 mo)	PCNSL protocol, CR Alive (44.7 mo)
13	M/74	4.0	Both	II	No	IV‐MTX	Corneal epitheliopathy	uCR	Yes (13.4 mo) Both	Yes (10.9 mo)	Salvage HD‐MTX, PD Expired (26.2 mo)
14	F/67	5.3	Left	II	No	IV‐MTX + HD‐MTX		CR	Yes (53.4 mo) Left	Yes (21.3 mo)	PCNSL protocol, CR Alive (65.0 mo)
15	F/79	13.1	Left	I	No	IV‐MTX		PR	Yes (7.2 mo) Left	No	PR after IV‐MTX, Alive (30.2 mo)
16	M/59	15.9	Left	I	No	IV‐MTX + HD‐MTX	Glaucoma and cataract	CR	Yes (16.1 mo) Both	No	CR after IV‐MTX, Alive (26.3 mo)
17	F/65	30.6	Right	I	No	IV‐MTX	Corneal epitheliopathy	CR	Yes (24.5 mo) Right	No	CR after IV‐MTX, Alive (68.6 mo)
18	F/56	4.3	Right	II	No	IV‐MTX	Corneal epitheliopathy Blepharitis and Cataract	CR	Yes (33.1 mo) Right	No	CR after IV‐MTX, Alive (54.4 mo)
19	F/69	13.7	Left	II	No	IV‐MTX	Corneal epitheliopathy	CR	Yes (36.7 mo) Right	No	CR after IV‐MTX, Alive (46.1 mo)
20	F/78	17.8	Right	I	No	IV‐MTX		CR	Yes (38.0 mo) Both	No	CR after IV‐MTX, Alive (25.5 mo)
21	M/57	8.7	Left	II	No	IV‐MTX	Corneal epitheliopathy	CR	Yes (53.4 mo) Left	No	CR after IV‐MTX, Alive (65.0 mo)

Abbreviations: CNS, central nervous system; CR, complete remission; HD‐MTX, high‐dose intravenous methotrexate; HSCT, hematopoietic stem cell transplantation; IL, interleukin; IV‐MTX, intravitreal methotrexate; PR, partial remission; R‐CHOP, consists of rituximab; cyclophosphamide; doxorubicin, vincristine; and prednisone; uCR, unconfirmed complete remission; WBRT, whole‐brain radiotherapy.

^a^ One patient relapsed as systemic DLBCL involvement (Ann‐Arbor stage IV), including CNS, required R‐CHOP chemotherapy.

Although the planned first‐line treatment was IV‐MTX combined with systemic therapy, Cases 1, 3, 5, 6, 7, 8, 13, 15, 17, 18, 19, 20, and 21 did not receive systemic HD‐MTX initially and were only treated with outpatient IV‐MTX injection regularly at the ophthalmology clinic. The comparison of clinical characteristics and treatment response between the IV‐MTX only (*n* = 13) and IV‐MTX with systemic treatment (*n* = 8) groups is shown in Table 3. There were no significant between‐group differences except for the higher number of patients with poor performance status (ECOG PS ≥2). Furthermore, there was no significant factor related to a cumulative incidence of CNS relapse in multivariate analysis. However, early treatment within 3 months from symptom onset (*n* = 12) presented a protective effect trend (HR 0.19, 95% CI, 0.04–1.04, *p* = 0.051) (Table S1).

**TABLE 3:** Comparison of clinical characteristics and treatment response between two different treatment groups (*N* = 24 21)

Category	IV‐MTX only (*n* = 13)	IV‐MTX with Systemic treatment (*n* = 8)	*p‐*Value
Age	66.38 ± 13.17 years	60.00 ± 7.17 years	0.225
Male sex	5 (38.5%)	6 (75.0%)	0.183
ECOG PS ≥2	1 (7.7%)	4 (50.0%)	0.047
Initial LDH, average	462.38 ± 86.79 IU	466.00 ± 135.35 IU	0.941
Diagnosis			
Level I	4 (30.8%)	3 (37.5%)	0.123
Level II	9 (69.2%)	3 (37.5%)	
Level III	0 (0%)	2 (25.0%)	
From symptom onset to diagnosis, average	11.6 ± 8.5 months	9.2 ± 6.4 months	0.483
Early treatment from symptom onset (≤ 3 months)	1 (7.7%)	2 (25.0%)	0.531
Treatment response, CR/uCR	12 (92.3%)	6 (75.0%)	0.531
Ocular relapse	7 (53.8%)	5 (62.5%)	1.000
CNS relapse	3 (23.1%)	5 (62.5%)	0.164

Abbreviations: CNS, central nervous system; CR, complete remission; ECOG PS, Eastern Cooperative Oncology Group performance status; IV‐MTX, intravitreal methotrexate; LDH, lactate dehydrogenase; uCR, unconfirmed complete remission.

### 3.3 Surveillance of the vitreous fluid IL‐10/IL‐6 cytokine ratio

**FIGURE 3:**
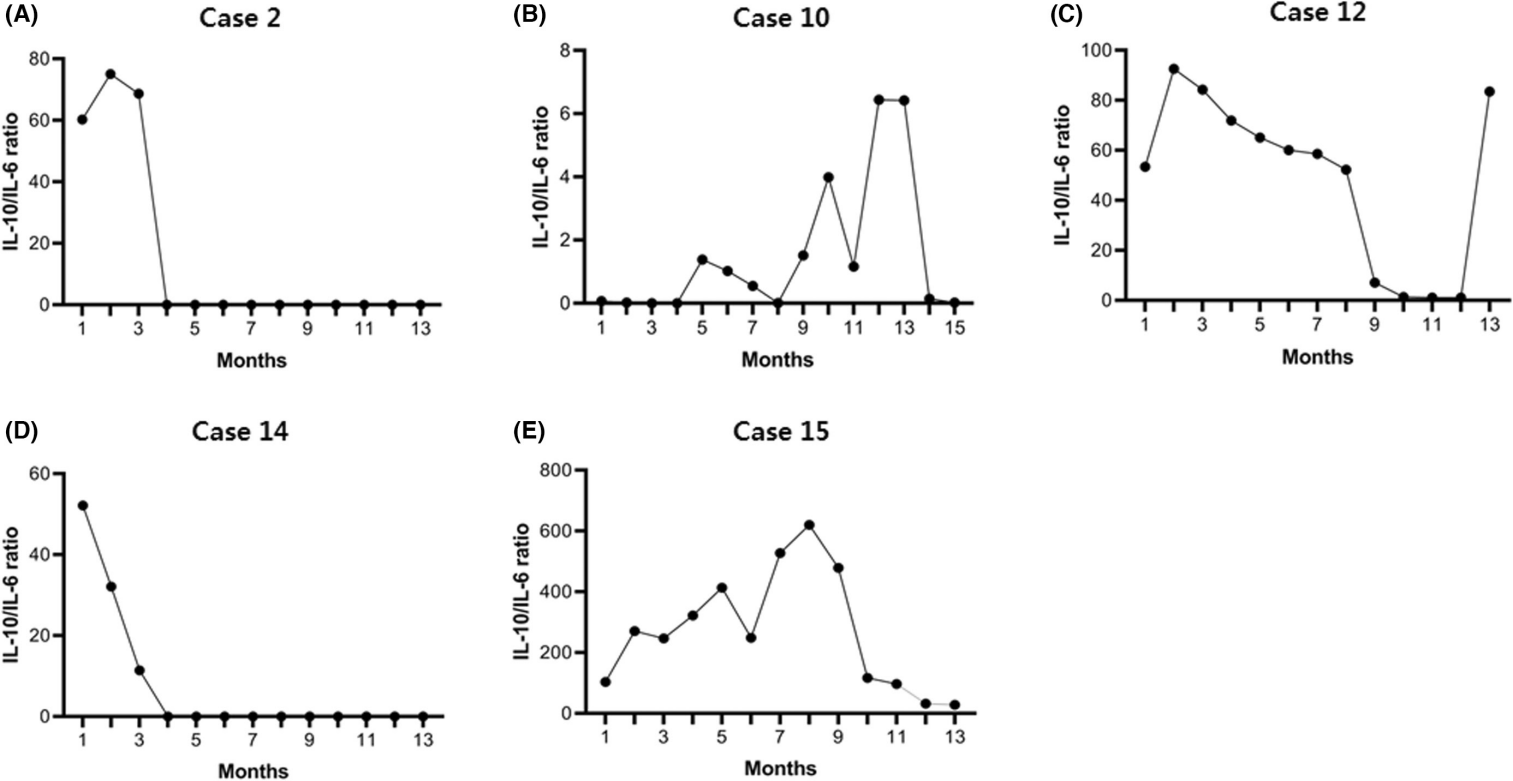
Clinical data of vitreous fluid IL‐10/IL‐6 ratio evaluation (A) Case 2 demonstrate a sustained reduction in the cytokine ratio (<1) without evidence of relapse in long‐term follow‐up. (B and C) Cases 10 and 12 show rapid elevation of the cytokine ratio before ophthalmologic and CNS relapse. (D) The vitreous fluid cytokine study of Case 14 was performed after identifying ocular relapse, and the cytokine ratio shows a gradual decrease after salvage treatments. (E) Case 15 refused HD‐MTX treatment and was only treated with IV‐MTX at a regular outpatient clinic. Although the cytokine ratio did not decrease below 1, this patient shows partial remission by ocular examination without specific visual symptoms or the occurrence of CNS lesions. CNS, central nervous system; HD‐MTX, high‐dose methotrexate; IV, intravenous.

Figure 3 shows the long‐term surveillance outcomes of the vitreous fluid IL‐10/IL‐6 cytokine ratio. Case 2 demonstrated a sustained reduction in the cytokine ratio (<1) without evidence of relapse in long‐term follow‐up. Cases 10 and 12 demonstrated rapid elevation of the cytokine ratio before ophthalmologic and CNS relapse; these patients currently receive salvage treatment, and their cytokine ratios are regularly monitored. The vitreous fluid cytokine study of Case 14, performed after diagnosing ophthalmologic relapse, showed a gradual decrease in the cytokine ratio after salvage treatments. This patient achieved CR on ocular examination. Case 15 refused systemic HD‐MTX treatments and only received regular IV‐MTX treatment. Although the cytokine ratio did not decrease below 1, this patient showed PR on ocular examination without specific visual symptoms or the occurrence of CNS lesions.

### 3.4 Adverse events

All 21 patients developed conjunctival hyperemia, and seven (33.3%) developed some form of keratopathy, ranging from diffuse punctate keratopathy to severe corneal epitheliopathy after the fifth injection. In four of the seven patients, this subsided completely after a reduction of the IV‐MTX injection to once a month, and in one patient, it began to subside after 2 weeks of injection rest. However, for two of the more severe cases, the injection was temporarily terminated for 2 months, and autologous serum eye drops treatment was administered. Complications included uncontrolled glaucoma (*n* = 3, 14.3%), acceleration of existing cataract that required surgery (*n* = 3, 14.3%), and blepharitis (*n* = 1, 4.8%). Although temporal increases in IOP immediately after IV‐MTX injection could be treated with digital massage therapy, three patients who experienced uncontrolled IOP elevation during IV‐MTX protocol needed trabeculectomy. The injections were postponed in one patient who developed macular edema with vitreous hemorrhage, and a bevacizumab injection was administered. One patient developed limbic ischemia and responded well to autologous serum eye drops (Figure 3).

## 4 DISCUSSION

This retrospective single‐center study including 21 PIOL patients was conducted using CULG (Catholic University Lymphoma Group) multidisciplinary care by skilled ophthalmologists specialized on the cornea, pathologists and hematologists specialized in lymphoma, and radiologists. Among the 21 patients, 29.2% (*n* = 7) were diagnosed via confirmative cytology and immunohistochemical staining, 57.2% (*n* = 12) via additional flow cytometry and molecular studies, and 9.5% (*n* = 2) via IL‐10/IL‐6 cytokine ratio using ELISA. However, given the “masquerade syndrome” nature of PIOL, the median interval between the onset of ocular symptoms and the diagnosis was delayed up to the median of 10.8 months. After PIOL diagnosis, eight patients underwent IV‐MTX with additional six cycles of systemic HD‐MTX to prevent CNS relapse and 13 with only IV‐MTX. There were no differences in demographic characteristics between the two groups, except that a higher number of patients who received IV‐MTX and HD‐MTX had poorer performance status scores compared to those receiving IV‐MTX alone. Furthermore, 12 ophthalmologic and eight CNS relapse cases within a median of 20.3 and 11.6 months were shown regardless of the treatment modalities, with a median PFS of 21.3 months. Because of frequent PIOL relapse in the CNS approximately 1 year after treatment and ophthalmologic lesions approximately 2 years after, regular clinical follow‐up is crucial, and utilizing the IL‐10/IL‐6 cytokine ratio level is recommended.^18^

To the best of our knowledge, the standard treatment strategy for PIOL is yet to be established. Furthermore, the preventive effect of prophylactic treatment, systemic chemotherapy, or radiotherapy on the ocular lesion or brain for future CNS lesions remains controversial. Vitrectomy and IV‐MTX injection improve visual symptoms by eliminating opacities formed by cellular debris and clumps in the vitreous cavity. However, they are not sufficient to prevent local ophthalmologic relapse or the occurrence of CNS lesions in the long term. Thus, we actively use systemic HD‐MTX intravenous infusion treatment and IV‐MTX to prevent CNS lesions. We also attempted to reduce the number of IV‐MTX injections to avoid various ocular damages and ophthalmic complications. The reason for selecting HD‐MTX as a prophylactic regimen was that unnecessary intensive treatment might induce chemotherapy‐related adverse events and worsen the patients' performance status without any profound preventive benefits. The related adverse outcomes might even limit the feasible treatment strategies in instances where CNS lesions develop.

Unfortunately, despite adding HD‐MTX treatment, this study showed no preventive effect in either ophthalmologic or CNS relapse. Furthermore, the discrepancy between OS and PFS could be explained using more effective treatment strategies than the frontline treatment, although overall, only half of the patients achieved CR after salvage therapy, thus highlighting the aggressiveness of PIOL. These findings suggest that the first‐line treatment modality for a patient with isolated PIOL should be more intensive, equivalent to PCNSL treatment protocol as 1 day of HD‐MTX (3.5 g/m^2^) and two consecutive days of cytarabine (3.0 g/m^2^/day).^21^ A recently published retrospective multicenter study also presented a similar conclusion that primary HD‐MTX‐based chemotherapy was feasible to control PIOL with an acceptable safety profile, but the rate of CNS or ocular relapse was high and highlighted the potential role of additional intensive treatment for this aggressive disease.^22^ Furthermore, intravitreal rituximab injection could also be considered an alternative for local treatment of PIOL. Intravenous rituximab has already been effective in CD20‐positive PCNSL systemic lymphoma and has been increasingly investigated in recent years as an acceptable treatment for PIOL. Moreover, its lower toxicity could be primarily for MTX‐resistant or relapsed PIOL.^23^, ^24^, ^25^ However, we did not have an opportunity to administer intravitreal rituximab treatment, which was considered to have arbitrary uninsured medical benefits, and illegal, in Korea.

The treatment of patients with CNS relapse in PIOL should follow the PCNSL treatment protocol: multicycle therapy with five to seven cycles of intravenous HD‐MTX with additional drugs (e.g., cytarabine or rituximab, procarbazine, and vincristine).^19^, ^21^, ^26^ Auto‐HSCT is an essential component of myeloablative chemotherapy in patients who achieved CR from systemic chemotherapy.^26^ Although WBRT has high antitumor activity, it has delayed neurotoxicity, especially in old‐aged patients, which results in significant morbidity and mortality.^21^ Therefore, WBRT should be reserved for salvage treatment after CNS relapse for either poor performance status or decreased renal function. In this study, many PIOL patients were either relatively old (*n* = 12, 57.2% were aged >60 years) or had poor performance status at diagnosis (*n* = 5, 23.8% had an ECOG PS ≥2). Therefore, most patients were not eligible for auto‐HSCT, and thus, we utilized the PCNSL protocol without transplantation, salvage WBRT, or HD‐MTX single regimen after CNS relapse.

IL‐10 is a growth and differentiation factor for B‐cell lymphomas and could be detected in the vitreous fluid of PIOL patients.^27^ The level of IL‐10 is strongly correlated with both clinical activity and numbers of malignant cells on cytopathology.^27^ Considering the difficulty of diagnosing PIOL due to the lack of characteristic features, the IL‐10 level and IL‐10/IL‐6 cytokine ratio measured via ELISA should be included in the routine diagnostic protocol for PIOL. In addition, because PIOL patients often experience distant relapse, an elevated level of IL‐10/IL‐6 cytokine ratio (>1) in the vitreous fluid during follow‐up could be a valuable indicator of relapse. Therefore, high‐risk patients should undergo stringent ophthalmologic evaluation at 3‐month intervals after the first year of follow‐up. Further, the IL‐10/IL‐6 ratio should be measured to provide helpful evidence for evaluating biochemical relapse.

This study has some limitations because of the retrospective design and small sample size. First, the treatment responses and visual symptom changes were not firmly assessed in a prospective study; thus, they may not be reliable or generalizable to other populations and there remains possible bias. Second, because of the ability of PIOL to mimic other ocular syndromes, many patients pretreated with other drugs, mostly corticosteroids, enrolled in this study (*n* = 12, 57.1%). Therefore, the cytolytic effect of corticosteroids on lymphoma cells probably has a negative impact on the diagnostic yield in the cytologic specimen for a definite diagnosis. Furthermore, the cytokine study (IL‐10/IL‐6 ratio in vitreous fluid) was unavailable until 2018 in this institution; thus, long‐term follow‐up data were limited in many patients. Third, there were few significant statistical analysis results, primarily because of a limited number of patients due to the rarity of the disease and relatively short follow‐up periods, which limited our ability to analyze PIOL prognostic factors. Finally, we did not have considerable experience in ocular external beam radiotherapy because of its potential yet permanent complications, such as radiation keratopathy, dry eyes, cataracts, radiation retinopathy, or papillopathy, which negatively affect the quality of life of PIOL patients and cannot be ignored.

In summary, stepwise diagnosis of PIOL by the depth of evidence might be an effective method to diagnose this confusable disease. Furthermore, the IL‐10/IL‐6 ratio in vitreous fluid evaluated by ELISA is essential to diagnose PIOL along with proficient clinical evidence and to assess prompt relapse by regular basis follow‐up. Unlike recently reported similar studies,^18^, ^28^ we experienced either ophthalmologic or CNS relapse frequently.^22^ Although frontline systemic HD‐MTX therapy did not prevent or delay CNS relapse in this study, the introduction of more intensive or even novel front‐line therapeutic regimens without systemic toxicity should be considered to avoid ophthalmologic and PIOL relapse in the CNS. Therefore, multicenter prospective study designs with a large cohort will impart the clinical benefits of systemic CNS prophylaxis protocols, intravitreal rituximab, combined regimen intravitreal and systemic chemotherapy, and individualized targeted therapy with novel agents.

## AUTHOR CONTRIBUTIONS

**Gi‐June Min:** Conceptualization (lead); data curation (lead); formal analysis (lead); methodology (lead); writing – original draft (lead). **Tong Yoon Kim:** Data curation (equal); methodology (equal); validation (supporting); visualization (lead). **Young‐Woo Jeon:** Conceptualization (equal); data curation (equal); formal analysis (equal); resources (supporting); supervision (supporting); validation (supporting); writing – review and editing (supporting). **Joo Hyun O:** Methodology (equal); supervision (supporting); validation (supporting); visualization (supporting); writing – review and editing (supporting). **Byung‐Ock Choi:** Methodology (supporting); supervision (supporting); writing – review and editing (supporting). **Gyeongsin Park:** Methodology (lead); writing – review and editing (supporting). **Young‐Hoon Park:** Conceptualization (equal); investigation (equal); methodology (equal); resources (equal); supervision (supporting); visualization (lead); writing – review and editing (lead). **Seok‐Goo Cho:** Conceptualization (equal); resources (supporting); supervision (lead); validation (lead); writing – review and editing (lead).

## FUNDING INFORMATION

The authors have no financial or proprietary disclosure to make in relation to this article.

## CONFLICT OF INTEREST

The authors declare that the research was conducted in the absence of any commercial or financial relationships that could be construed as a potential conflict of interest.

## ETHICAL APPROVAL

This retrospective study was approved by the Institutional Review Board and Ethics Committee of the Catholic Medical Center, Republic of Korea (KC21RASI0761) and conducted according to the tenets of the Helsinki Declaration.

## Supporting information


Data S1

## Data Availability

The data that support the findings of this study are available upon request from the corresponding author. The data are not publicly available because of privacy or ethical restrictions.
