# Supplementary material for: Diagnosis, treatment, and prognosis of primary intraocular lymphoma: Single‐center real‐world clinical experience
Source: Cancer Med. 2023 Jan 31;12(7):7911–22. doi: 10.1002/cam4.5567 (PMC10134376; doi:10.1002/cam4.5567)
Supplement: Supplementary file 1 — Data S1 [file CAM4-12-7911-s001.docx]

**Supporting Information**

**Supplementary Table S1. Univariate and multivariate analyses for incidence of CNS relapse**

| **Variables** | **Cumulative incidence of CNS relapse** | ***p-value*** | **HR (95% CI)** | ***p-value*** |
| --- | --- | --- | --- | --- |
| **Age** |  | 0.354 |  |  |
| < 60 years (n = 8) | 28.6% (3.1–63.6) |  |  |  |
| ≥ 60 years (n = 13) | 49.6% (18.4–74.8) |  |  |  |
| **Sex** |  | 0.389 |  |  |
| Male (n = 11) | 50.0% (16.1–76.9) |  |  |  |
| Female (n = 10) | 32.9% (6.4–63.8) |  |  |  |
| **ECOG** |  | 0.266 |  |  |
| 0–1 (n = 16) | 32.8% (11.1–56.8) |  |  |  |
| ≥ 2 (n = 5) | 75.0% (1.7–98.0) |  |  |  |
| **Initial LDH** |  | 0.191 |  |  |
| Normal (n = 10) | 25.7% (3.0–59.2) |  |  |  |
| Elevated (n = 11) | 54.5% (20.4–79.4) |  |  |  |
| **Diagnosis level** |  | 0.298 |  |  |
| Level I (n = 7) | 28.6% (2.9–64.1) |  |  |  |
| Level II (n = 12) | 44.4% (14.4-71.2) |  |  |  |
| Level III (n = 2) | 100% |  |  |  |
| **Initial treatment** |  | 0.096 | 2.05 (0.47–8.85) | 0.340 |
| IV–MTX only (n = 13) | 23.9% (5.1–50.2) |  |  |  |
| IV–MTX with systemic treatment (n = 8) | 71.4% (14.9–94.2) |  |  |  |
| **Pretreatment** |  | 0.235 |  |  |
| No (n = 9) | 55.0% (11.2–84.9) |  |  |  |
| Yes (n = 12) | 33.3% (9.2–60.3) |  |  |  |
| **Early treatment from symptom onset** |  | 0.026 | 0.19 (0.04–1.04) | 0.051 |
| > 3 months (n = 9) | 74.6% (19.8–94.8) |  |  |  |
| ≤ 3 months (n = 12) | 18.3% (2.5–45.9) |  |  |  |
| **Treatment response** |  | 0.332 |  |  |
| CR/uCR (n = 18) | 37.2% (14.4–60.5) |  |  |  |
| PR (n = 3) | 66.7% (0.2–97.3) |  |  |  |

CR, complete remission; CI, confidence interval; CNS, central nervous system; HR, hazard ratio; IV-MTX, intravitreal methotrexate; LDH, lactate dehydrogenase; PR, partial remission; uCR, unconfirmed complete remission

| 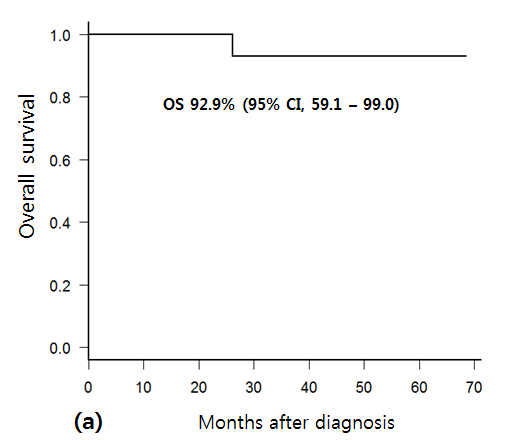 | 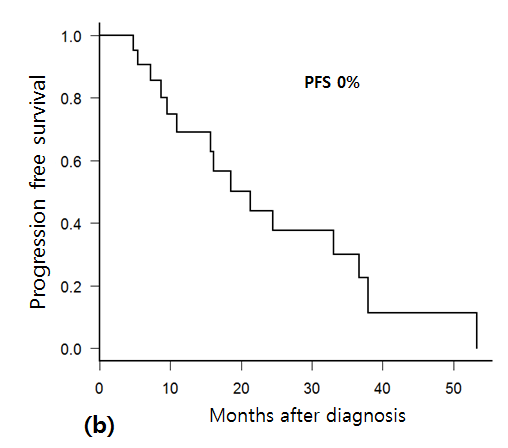 |
| --- | --- |
| 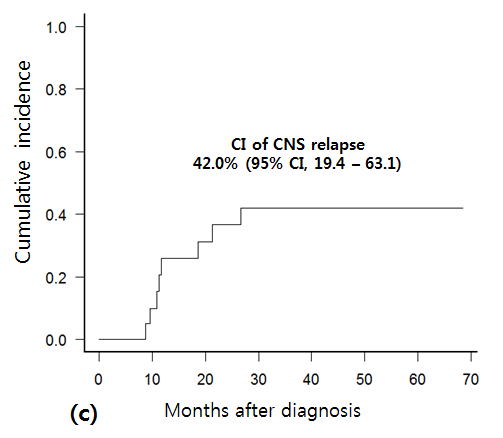 | 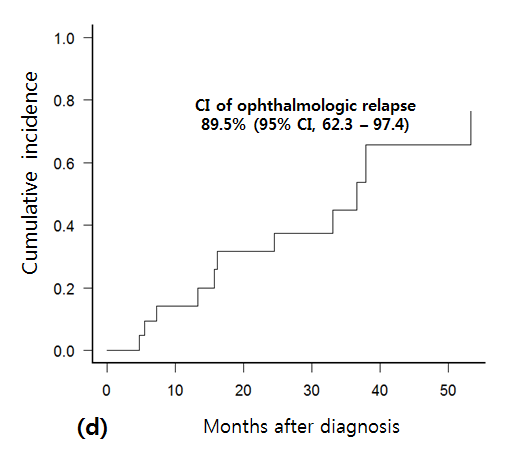 |

**Supplementary Figure S1.** Survival outcomes of patients with primary intraocular lymphoma (**a**) Overall survival (OS); (**b**) Progression-free survival (PFS); (**c**) Cumulative incidence of central nervous system relapse (CIR of CNS); (**d**) Cumulative incidence of ophthalmologic relapse (ophthalmologic CIR). CI, confidence interval
